# Evaluation of the role of atherogenic index of plasma in the reversion from Prediabetes to normoglycemia or progression to Diabetes: a multi-center retrospective cohort study

**DOI:** 10.1186/s12933-023-02108-8

**Published:** 2024-01-06

**Authors:** Hongyi Yang, Maobin Kuang, Ruijuan Yang, Guobo Xie, Guotai Sheng, Yang Zou

**Affiliations:** 1 Department of Ultrasound, the Second Affiliated Hospital of Nanchang University, Nanchang, Jiangxi Province 330006 P.R. China; 2 Department of Internal Medicine, Jiangxi Medical College, Nanchang University, Nanchang, Jiangxi Province 330006 P.R. China; 3 Jiangxi Cardiovascular Research Institute, Jiangxi Provincial People’s Hospital, The First Affiliated Hospital of Nanchang Medical College, Nanchang, Jiangxi Province 330006 P.R. China; 4 Department of Endocrinology, Jiangxi Provincial People’s Hospital, The First Affiliated Hospital of Nanchang Medical College, Nanchang, Jiangxi Province 330006 P.R. China; 5 Jiangxi Provincial Geriatric Hospital, Jiangxi Provincial People’s Hospital, The First Affiliated Hospital of Nanchang Medical College, Nanchang, Jiangxi Province 330006 P.R. China

## Abstract

**Background:**

Atherosclerosis is closely linked with glucose metabolism. We aimed to investigate the role of the atherogenic index of plasma (AIP) in the reversal of prediabetes to normal blood glucose levels or its progression to diabetes.

**Methods:**

This multi-center retrospective cohort study included 15,421 prediabetic participants from 32 regions across 11 cities in China, under the aegis of the Rich Healthcare Group’s affiliated medical examination institutions. Throughout the follow-up period, we monitored changes in the glycemic status of these participants, including reversal to normal fasting glucose (NFG), persistence in the prediabetic state, or progression to diabetes. Segmented regression, stratified analysis, and restricted cubic spline (RCS) were performed based on the multivariable Cox regression model to evaluate the association between AIP and the reversal of prediabetes to NFG or progression to diabetes.

**Results:**

During a median follow-up period of 2.9 years, we recorded 6,481 individuals (42.03%) reverting from prediabetes to NFG, and 2,424 individuals (15.72%) progressing to diabetes. After adjusting for confounders, AIP showed a positive correlation with the progression from prediabetes to diabetes [(Hazard ratio (HR) 1.42, 95% confidence interval (CI):1.24–1.64)] and a negative correlation with the reversion from prediabetes to NFG (HR 0.89, 95%CI:0.81–0.98); further RCS demonstrated a nonlinear relationship between AIP and the reversion from prediabetes to NFG/progression to diabetes, identifying a turning point of 0.04 for reversion to NFG and 0.17 for progression to diabetes. In addition, we observed significant differences in the association between AIP and reversion from prediabetes to NFG/progression to diabetes across age subgroups, specifically indicating that the risk associated with AIP for progression from prediabetes to diabetes was relatively higher in younger populations; likewise, a younger age within the adult group favored the reversion from prediabetes to NFG in relation to AIP.

**Conclusion:**

Our study, for the first time, reveals a negative correlation between AIP and the reversion from prediabetes to normoglycemia and validates the crucial role of AIP in the risk assessment of prediabetes progression. Based on threshold analysis, therapeutically, keeping the AIP below 0.04 was of paramount importance for individuals with prediabetes aiming for reversion to NFG; preventatively, maintaining AIP below 0.17 was vital to reduce the risk of diabetes onset for those with prediabetes.

**Supplementary Information:**

The online version contains supplementary material available at 10.1186/s12933-023-02108-8.

## Background

Prediabetes is a high-risk state for diabetes, as well as various vascular-related diseases and chronic conditions [1–4]. Extensive research in the past has assessed the progression of prediabetes, yielding significant findings that have greatly influenced diabetes prevention policies [5–7]. However, the regression of prediabetes has recently garnered attention, with completed randomized controlled trials of pharmacological or lifestyle interventions suggesting that reversing prediabetes can significantly protect patients from future diabetes and various chronic complications [8–19]. Given the current global pandemic of prediabetes, with prediabetic patients exceeding 400 million, and potential widespread complications [1–4, 20], it’s crucial to actively explore modifiable factors beneficial for the reversion or progression of prediabetes.

AIP is a simple parameter employed for assessing plasma atherosclerosis, first proposed by Professors Frohlich J and Dobiásová M in 2001 [21]. In their initial investigation, Professor Frohlich J and his team examined the relationship between AIP and the fractional esterification rate of high-density lipoprotein cholesterol, as well as lipoprotein particle size. They found that AIP values closely resembled the fractional esterification rate of high-density lipoprotein cholesterol and lipoprotein particle size, suggesting AIP as a potentially useful simple parameter representing atherosclerosis [21]. This proposition was further validated in subsequent clinical studies, with many researchers also identifying the significant value of AIP not only in evaluating atherosclerosis-related diseases and their adverse outcomes [22–28], but also in reflecting insulin resistance (IR), which is closely linked to glucose metabolic dysfunction [29, 30]. Recent observational studies have further corroborated the significant role of AIP in the onset of prediabetes and diabetes [31–33]. However, the impact of AIP on blood glucose reversal or progression in individuals with prediabetes remains unclear. Given the high prevalence of prediabetes and the severe physical damage caused by its widespread complications, determining the relationship between the modifiable factor, AIP, and glycemic status changes in prediabetic individuals may offer significant benefits in reversing this situation. Hence, in the present study, based on the multi-center medical examination data from China’s Rich Healthcare Group, we aimed to explore the role of AIP in the reversal of prediabetes to NFG or its progression to diabetes.

## Methods

### Study design and data source

This study utilized the longitudinal follow-up data from the multi-center health examination cohort of Rich Healthcare Group. The establishment of this dataset was initially aimed at investigating the significant role of obesity in the onset of diabetes among the Chinese population. The detailed design has been previously described elsewhere by Professor Li Xiaoying and colleagues [34]. In brief, in the original design, Li et al. initially enrolled 685,277 adult participants who underwent health screenings at Rich Healthcare Group health examination centers across 32 districts in 11 cities in China between 2010 and 2016, and these participants had at least two or more health screenings during this period. In line with the initial research objectives, Li et al. excluded participants with missing baseline information [including sex, age, and fasting plasma glucose (FPG)] (n = 135,317), those with body mass index (BMI) values above 55 kg/m^2^ or below 15 kg/m^2^ (n = 152), those with a follow-up duration less than 2 years (n = 324,233), those diagnosed with diabetes at baseline (n = 7,112), and participants whose diabetes status could not be determined during follow-up (n = 6,630). Ultimately, they incorporated 211,833 participants into the study and completed the entire research. The corresponding dataset has been anonymized by Professor Li’s team and shared publicly on the DRYAD database [35]. In accordance with the terms of use of the DRYAD database and the Creative Commons Attribution-NonCommercial-ShareAlike 4.0 International License (CC BY-NC-ND 4.0), researchers can utilize datasets from the DRYAD database for secondary creation and must acknowledge the data source [35].

**Fig. 1:**
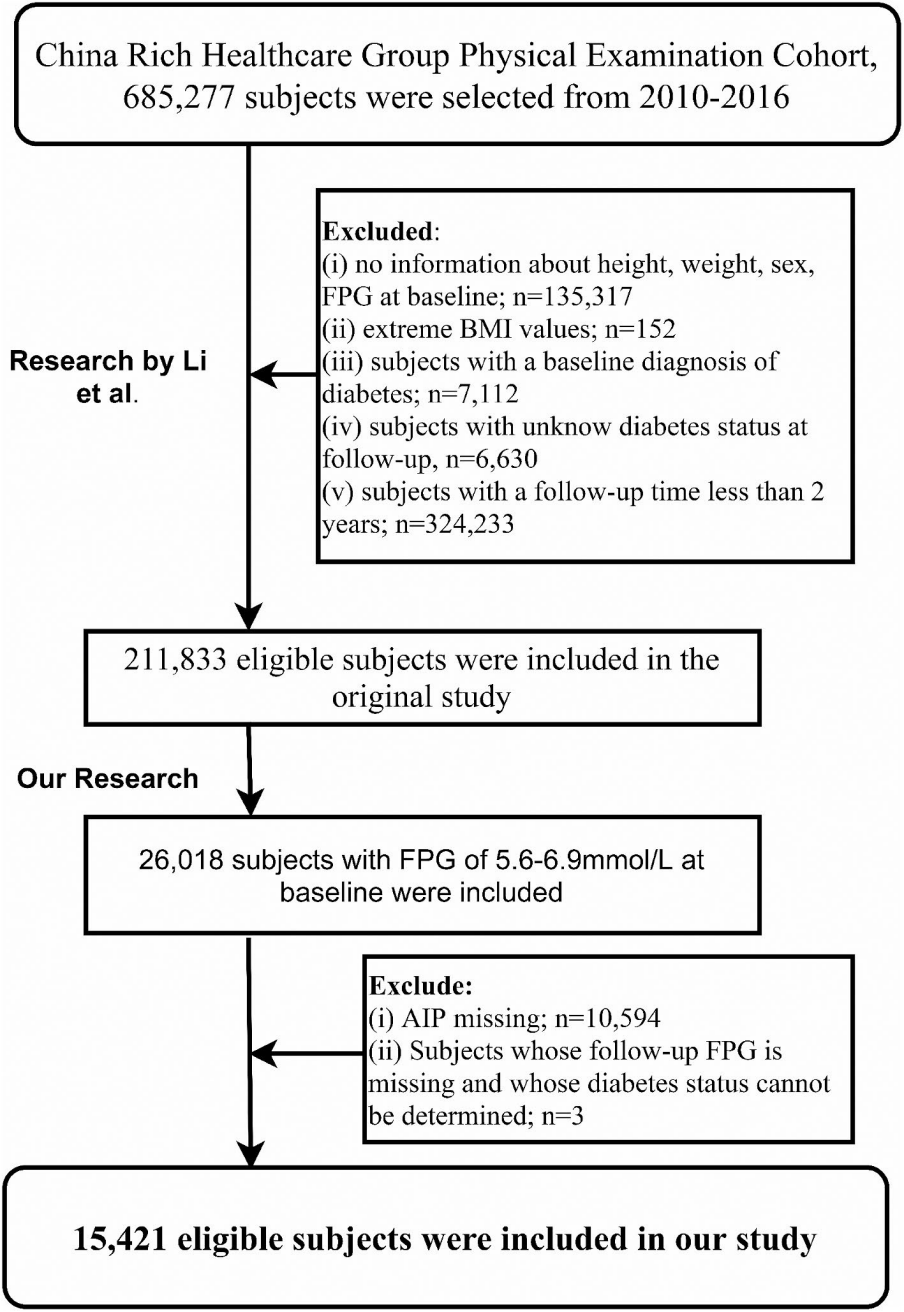
Flow chart of study participants

According to our research design, we initially identified individuals with baseline prediabetes in the dataset provided by Li et al. as the study population and set the AIP as the independent variable. The dependent variables were defined as changes in glycemic status during the follow-up, which includes reversion to NFG and progression to diabetes from prediabetes. Based on our new research objectives, we further excluded participants with missing independent variable data and those missing FPG during the follow-up and who were unable to determine diabetes status. Ultimately, our study included 15,421 participants for the secondary analyses. Figure 1 summarizes the entire research workflow.

### Ethical approval

In accordance with local laws and regulations, the Institutional Ethics Committee of Jiangxi Provincial People’s Hospital, after reviewing the design of the current study and the anonymized research data set, authorized the implementation of the current study and exempted the subjects from signing informed consent forms. The current study was conducted in line with the Declaration of Helsinki and adhered to the STROBE reporting guidelines.

### Baseline indicators measurement and assessment

As previously mentioned [34], participants at the health examination center were received by trained medical personnel who collected general demographic information (sex, age), lifestyle factors (smoking and drinking status), history of diabetes, and family history of diabetes. Simple physical parameters, such as height, weight, and blood pressure [systolic blood pressure (SBP) and diastolic blood pressure (DBP)], were also measured and recorded using a standardized questionnaire. For the measurement of height and weight, participants were asked to remove their shoes and wear only light clothing, with the results recorded to one decimal place. Blood pressure was measured using a mercury sphygmomanometer.

After fasting for at least 10 h, venous blood samples were obtained from the participants. In a standard laboratory setting, common biochemical indicators such as FPG, total cholesterol (TC), triglycerides (TG), high-density lipoprotein cholesterol (HDL-C), low-density lipoprotein cholesterol (LDL-C), creatinine (Cr), blood urea nitrogen (BUN), alanine aminotransferase (ALT), and aspartate aminotransferase (AST) were measured using an automated analyzer (Beckman 5800).

### Calculation

BMI was calculated as Weight(kg)/Height(m)^2^;

AIP was calculated as log10 (TG/HDL-C) [21].

### Definition of outcome

The outcome of interest in the present study was the change in glycemic status of participants with prediabetes during the follow-up period, including progression to diabetes, continued maintenance of a prediabetic state, or reversion to NFG. The study’s outcome definitions were based on the American Diabetes Association’s standards for prediabetes and diabetes using FPG [36]. Specifically, diabetes was defined as either a self-reported diagnosis of diabetes by another healthcare professional during follow-up or a measured FPG greater than 7.0 mmol/L. Prediabetes was defined as FPG levels greater than 5.6 mmol/L but less than 6.9 mmol/L. Reversion to NFG was defined as FPG levels below 5.6 mmol/L during follow-up.

### Statistical analysis

The baseline characteristics of the study population were summarized by quartiles of the independent variable and the outcomes. Categorical variables were reported as frequencies (%) and continuous variables as medians (interquartile range) or mean (standard deviation). Differences between groups were tested using one-way ANOVA (for continuous variables) and the chi-square test (for categorical variables).

Multivariable Cox regression models were used to determine the impact of AIP on glycemic state transitions among prediabetic participants. For multi-class outcomes, we split the data into binary datasets for each class using the one-versus-one method [37, 38]. Before modeling, we plotted Schoenfeld residuals over time to test the proportional hazards assumption [39] and calculated the variance inflation factor to check for potential multicollinearity [40]. Based on the STROBE statement [41], we applied four stepwise adjusted models to assess the associations between AIP (and its quartiles) and prediabetic glucose state transitions: an unadjusted reference model was first established, then Model I adjusted for sex and age; Model II further adjusted for SBP, LDL-C, ALT, BUN, and Cr; Model III built upon Model II and further adjusted for BMI, smoking status, drinking status, and family history of diabetes. Notably, in all models, we also tested for linear trends by modeling the median value of AIP quartiles as a continuous variable. Additionally, we explored the potential heterogeneity of the AIP association with prediabetic glucose state transitions in the most common phenotypes, including BMI, age, and sex; with BMI categorization based on the recommendations of the Working Group on Obesity in China [42], and age grouping referencing the World Health Organization (WHO) criteria [43]; likelihood ratio tests were used for comparing differences between groups.

Several sensitivity analyses were conducted to test the robustness of our findings: (1) Similar association analysis steps were performed based on WHO criteria for prediabetes/diabetes using FPG [44]. (2) Given potential competing risks among study outcomes, we further validated the association between AIP and reversion/progression of prediabetes in a competing risk model. (3) The same association analysis was conducted among those without a family history of diabetes. (4) We also computed the E-value to quantify the required strength of an unmeasured confounder [45].

Additionally, we employed 4-knot RCS in the Cox regression model to fit the dose-response relationship between AIP and prediabetes reversion/progression. When a non-linear association was detected, potential inflection points were identified using a recursive algorithm, and segmented Cox regression was used to evaluate HRs of glycemic state transitions before and after these points.

All tests considered a significance level set at *P* < 0.05. All analyses were performed using R language version 3.4.3 and Empower(R) version 2.0.

## Results

### Baseline characteristics summarized according to AIP quartiles

Among the 15,421 adult participants who met the inclusion criteria, 10,009 were males and 5,412 were females, with an average age of 51 years. The AIP displayed a normal distribution in this cohort (as shown in Supplementary Fig. 1). The baseline characteristics of the study population were presented according to the quartiles of AIP. As illustrated in Table 1, participants with a higher AIP were predominantly male, had a higher prevalence of smoking and drinking habits, and exhibited elevated values in age, height, weight, BMI, and levels of FPG, TC, TG, ALT, AST, and Cr.

**Table 1:** Summary of baseline characteristics of the study population according to AIP quartile group

	AIP quartiles				*P*-value
	Q1(-1.40,-0.16)	Q2(-0.15,0.05)	Q3(0.05–0.25)	Q4(0.25–1.41)	
No. of subjects	3855	3852	3858	3856	
Age, years	48.00 (37.00–59.00)	51.00 (39.00–61.00)	52.00 (42.00–61.00)	52.00 (42.00–61.00)	< 0.001
Sex					< 0.001
Male	1835 (47.60%)	2416 (62.72%)	2743 (71.10%)	3015 (78.19%)	
Female	2020 (52.40%)	1436 (37.28%)	1115 (28.90%)	841 (21.81%)	
Height, cm	164.65 (8.22)	166.26 (8.44)	167.29 (8.33)	168.45 (8.03)	< 0.001
Weight, kg	62.50 (10.64)	68.29 (11.16)	71.63 (11.59)	74.67 (11.80)	< 0.001
BMI, kg/m^2^	22.98 (3.04)	24.63 (3.14)	25.51 (3.11)	26.22 (3.06)	< 0.001
SBP, mmHg	123.21 (17.63)	127.44 (17.57)	129.08 (17.46)	130.27 (17.33)	< 0.001
DBP, mmHg	75.30 (10.81)	77.96 (11.10)	79.72 (11.09)	81.05 (10.94)	< 0.001
FPG, mmol/L	5.90 (0.29)	5.93 (0.30)	5.98 (0.33)	6.01 (0.34)	< 0.001
TC, mmol/L	4.79 (0.89)	4.99 (0.91)	5.11 (0.93)	5.28 (0.98)	< 0.001
TG, mmol/L	0.75 (0.60–0.90)	1.21 (1.06–1.40)	1.72 (1.51-2.00)	2.90 (2.35–3.81)	< 0.001
HDL-C, mmol/L	1.53 (1.36–1.71)	1.38 (1.23–1.54)	1.26 (1.11–1.42)	1.08 (0.95–1.27)	< 0.001
LDL-C, mmol/L	2.72 (2.31–3.17)	2.91 (2.48–3.37)	2.99 (2.54–3.46)	2.88 (2.43–3.42)	< 0.001
ALT, U/L	16.30 (12.30–23.00)	21.00 (15.00-29.50)	24.00 (17.40–35.10)	29.00 (20.50–42.60)	< 0.001
AST, U/L	21.90 (18.00–26.00)	23.45 (19.72–28.28)	24.00 (20.60-29.17)	26.40 (21.90–32.10)	< 0.001
BUN, mmol/L	4.87 (4.10–5.72)	4.90 (4.12–5.80)	4.89 (4.12–5.74)	4.86 (4.10–5.70)	0.174
Cr, umol/L	67.00 (57.00-79.50)	72.80 (61.00–83.00)	75.00 (63.80–85.00)	75.50 (65.20–85.00)	< 0.001
Family history of diabetes	94 (2.44%)	102 (2.65%)	89 (2.31%)	99 (2.57%)	0.785
Smoking status					0.252
Current	320 (8.30%)	308 (8.00%)	324 (8.40%)	358 (9.28%)	
Past	65 (1.69%)	63 (1.64%)	63 (1.63%)	68 (1.76%)	
Never	908 (23.55%)	869 (22.56%)	877 (22.73%)	940 (24.38%)	
Not recorded	2562 (66.46%)	2612 (67.81%)	2594 (67.24%)	2490 (64.57%)	
Drinking status					0.095
Current	51 (1.32%)	47 (1.22%)	56 (1.45%)	61 (1.58%)	
Past	243 (6.30%)	199 (5.17%)	224 (5.81%)	241 (6.25%)	
Never	999 (25.91%)	994 (25.80%)	984 (25.51%)	1064 (27.59%)	
Not recorded	2562 (66.46%)	2612 (67.81%)	2594 (67.24%)	2490 (64.57%)	

Values were expressed as mean (standard deviation) or median (interquartile range) or n (%)

Abbreviations: AIP: atherogenic index of plasma; BMI: body mass index; SBP: systolic blood pressure; DBP: diastolic blood pressure; FPG: fasting plasma glucose; TG: triglyceride; TC: total cholesterol; HDL-C: high-density lipoprotein cholesterol; LDL-C: low-density lipoprotein cholesterol; ALT: alanine aminotransferase; AST: aspartate aminotransferase; BUN: blood urea nitrogen; Cr: creatinine

### Baseline characteristics summarized according to follow-up outcomes

**Fig. 2:**
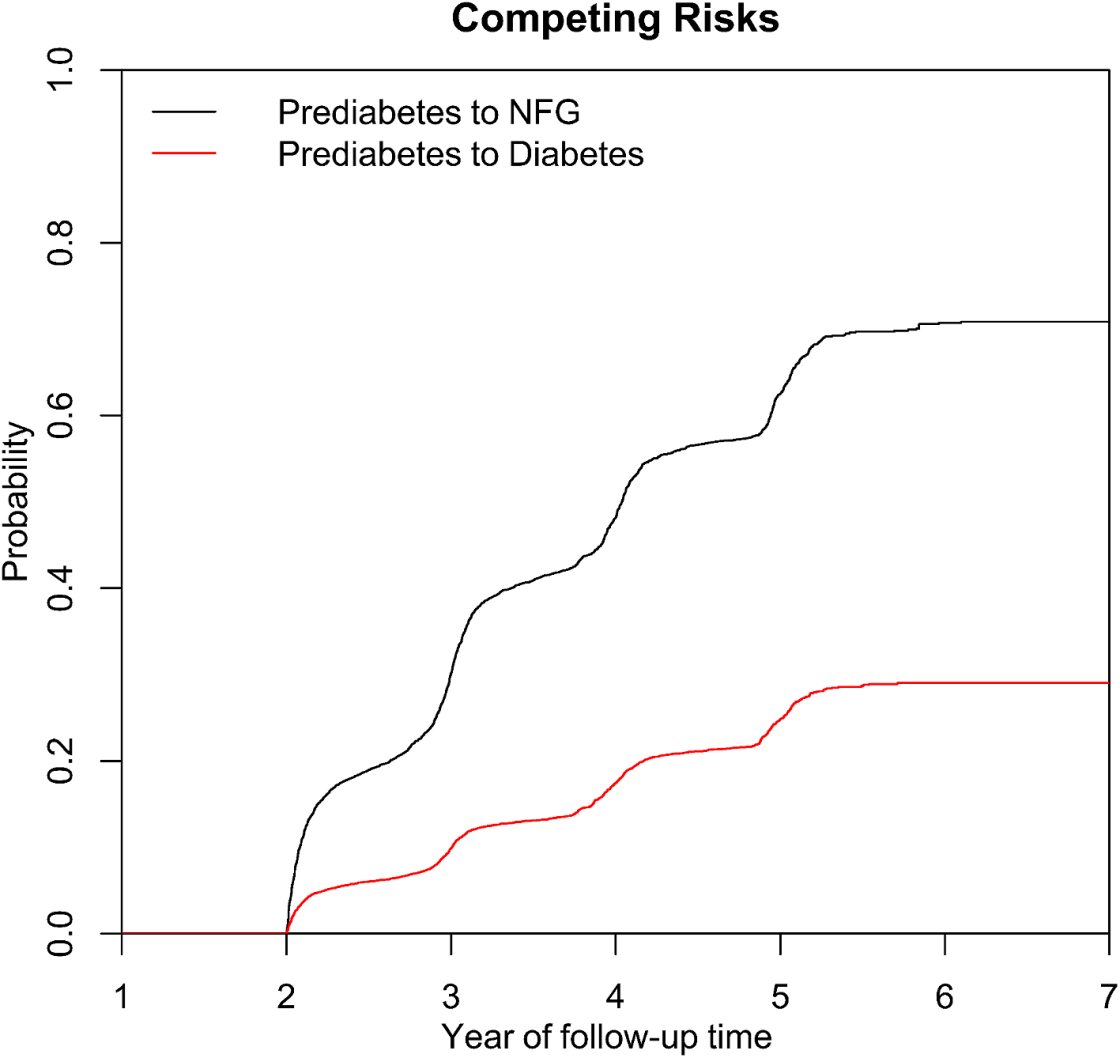
Cumulative incidence curve of reversal of prediabetes to NFG or progressing to diabetes. NFG: normal fasting glucose

During the median follow-up of 2.9 years, of the 15,421 participants, 2,424 (15.72%) developed new-onset diabetes, 6,516 (42.25%) remained in the prediabetic state, and 6,481 (42.03%) reverted to NFG. The incidence of prediabetes progressing to diabetes was 53 per 1,000 person-years (the incidence rates corresponding to AIP quartiles were 35 per 1000 person-years, 49 per 1000 person-years, 60 per 1000 person-years, and 68 per 1000 person-years, respectively), and reversing to NFG was 142 per 1,000 person-years (the incidence rates corresponding to AIP quartiles were 172 per 1000 person-years, 150 per 1000 person-years, 124 per 1000 person-years, and 121 per 1000 person-years, respectively). Figure 2 displays the cumulative incidence curves for the progression from prediabetes to diabetes and reversion to NFG, highlighting the significant potential for reversion from prediabetes to NFG.

**Fig. 3:**
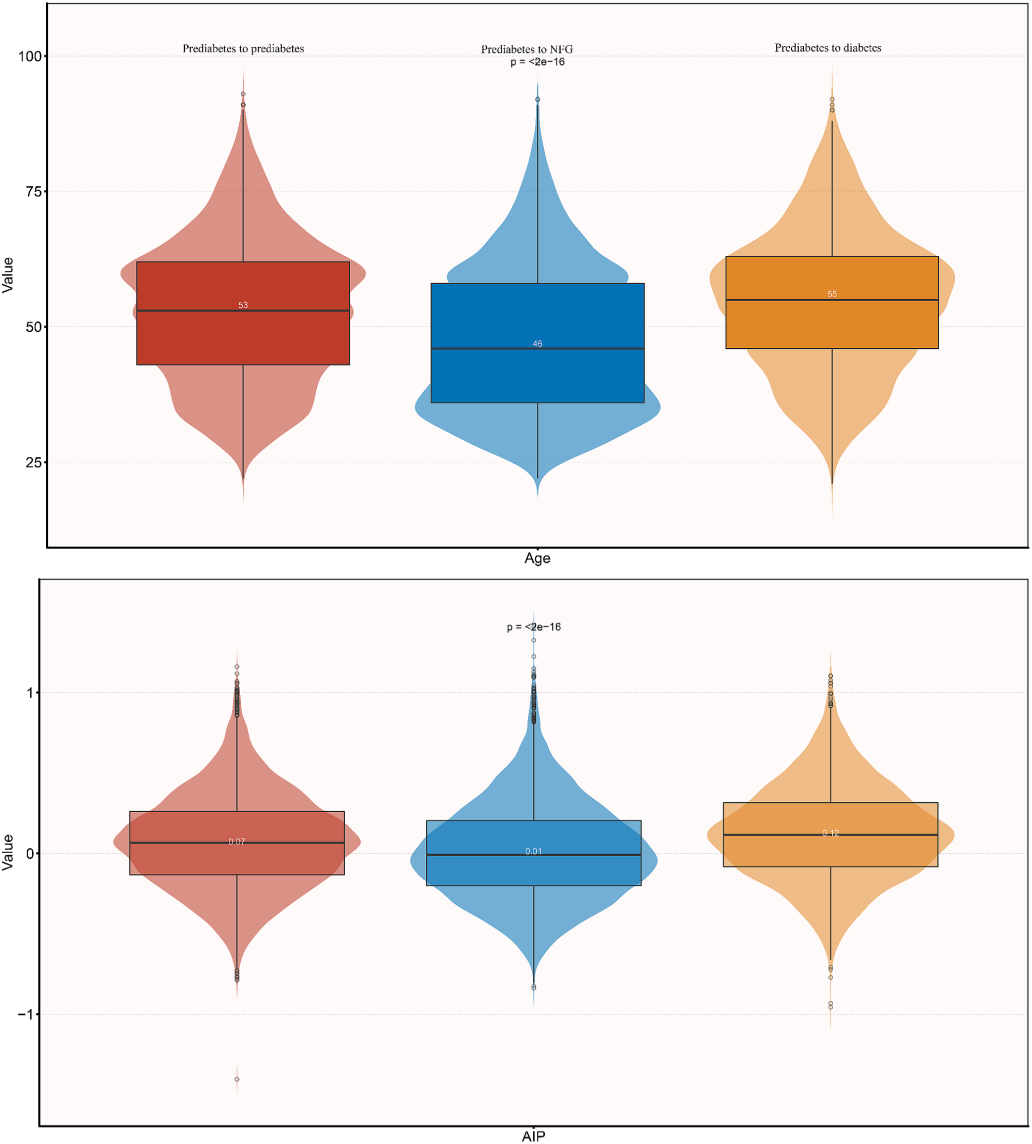
Violin chart showing baseline characteristics of AIP and age according to glucose status during follow-up. AIP: atherogenic index of plasma; NFG: normal fasting glucose

Pursuant to the follow-up outcomes, we further summarized the baseline characteristics of the participants. As indicated in Table 2, participants who eventually reverted to NFG typically had lower baseline measurements of age, weight, BMI, SBP, DBP, FPG, TC, TG, LDL-C, AIP, ALT, AST, BUN, and Cr, particularly age and AIP (see Fig. 3). Additionally, a greater proportion of this subgroup reported having quit smoking and had a lower prevalence of a family history of diabetes.

**Table 2:** Baseline characteristics summarized according to subjects’ glycemic status during follow-up

	Glucose status during follow-up			*P*-value
	Prediabetes	NFG	Diabetes	
No. of subjects	6516	6481	2424	
Sex				< 0.001
Male	4358 (66.88%)	3949 (60.93%)	1702 (70.21%)	
Female	2158 (33.12%)	2532 (39.07%)	722 (29.79%)	
Age, years	53.00 (43.00–62.00)	46.00 (36.00–58.00)	55.00 (46.00–63.00)	< 0.001
Height, cm	166.59 (8.37)	166.71 (8.40)	166.74 (8.31)	0.625
Weight, kg	69.80 (11.81)	67.64 (12.12)	72.22 (12.60)	< 0.001
BMI, kg/m^2^	25.06 (3.19)	24.23 (3.28)	25.87 (3.41)	< 0.001
SBP, mmHg	129.35 (17.76)	124.16 (16.93)	131.46 (17.99)	< 0.001
DBP, mmHg	79.53 (11.24)	76.73 (10.84)	80.49 (11.31)	< 0.001
FPG, mmol/L	6.00 (0.32)	5.84 (0.24)	6.15 (0.38)	< 0.001
TC, mmol/L	5.08 (0.94)	4.99 (0.94)	5.10 (0.97)	< 0.001
TG, mmol/L	1.50 (1.04–2.20)	1.31 (0.90–1.97)	1.67 (1.14–2.45)	< 0.001
HDL-C, mmol/L	1.31 (1.13–1.51)	1.34 (1.15–1.54)	1.29 (1.09–1.50)	< 0.001
LDL-C, mmol/L	2.90 (2.46–3.36)	2.84 (2.41–3.33)	2.88 (2.43–3.40)	< 0.001
AIP	0.07 (0.30)	0.01 (0.31)	0.12 (0.30)	< 0.001
ALT, U/L	22.50 (16.00–33.00)	20.40 (14.50–31.00)	24.90 (17.80–37.00)	< 0.001
AST, U/L	24.00 (20.00-28.80)	23.50 (19.40–28.80)	25.00 (20.70–31.00)	< 0.001
BUN, mmol/L	4.91 (4.19–5.78)	4.80 (4.05–5.68)	4.93 (4.13–5.80)	< 0.001
Cr, umol/L	73.50 (62.10–84.00)	72.00 (59.80–82.90)	73.30 (62.00–83.00)	< 0.001
Family history of diabetes	151 (2.32%)	148 (2.28%)	85 (3.51%)	0.002
Smoking status				< 0.001
Current	577 (8.86%)	491 (7.58%)	242 (9.98%)	
Past	94 (1.44%)	121 (1.87%)	44 (1.82%)	
Never	1471 (22.58%)	1642 (25.34%)	481 (19.84%)	
Not recorded	4374 (67.13%)	4227 (65.22%)	1657 (68.36%)	
Drinking status				0.005
Current	100 (1.53%)	86 (1.33%)	29 (1.20%)	
Past	342 (5.25%)	429 (6.62%)	136 (5.61%)	
Never	1700 (26.09%)	1739 (26.83%)	602 (24.83%)	
Not recorded	4374 (67.13%)	4227 (65.22%)	1657 (68.36%)	

Values were expressed as mean (standard deviation) or median (interquartile range) or n (%)

Abbreviations: NFG: normal fasting glucose; other abbreviations as in Table 1

### Association of AIP with glycemic state transition during the follow-up among prediabetic participants

Using reversion to NFG and progression to diabetes during the follow-up as dependent variables, we plotted the Schoenfeld residual plots for the AIP over time (as shown in Supplementary Figs. 2 and 3) and tested for collinearity between AIP and other covariates. The results indicated that the current study’s employment of the Cox model as the primary analytical method adheres to the proportional hazards assumption. Moreover, weight, TC, and TG, due to their variance inflation factor exceeding 5, were excluded from subsequent multivariable models (Supplementary Table 1).

Four Cox regression models were employed to assess the association between AIP and either reversion or progression of prediabetes (as summarized in Table 3). In the unadjusted model, we initially observed a negative association between AIP and reversion from prediabetes to NFG (HR 0.65, 95% CI:0.59–0.70) and a positive association between AIP and progression from prediabetes to diabetes (HR 2.07, 95%CI:1.83–2.35). Subsequently, in the sequentially adjusted multivariable Cox regression models (Models I-III), we noticed a partial attenuation in the strength of association between AIP and either reversion to NFG or progression to diabetes. However, the overall directional consistency of the association was retained from the initial model. Furthermore, we assessed the relationship between AIP quartiles and either reversion to NFG or progression to diabetes in all models. The results indicated that as quartiles of AIP increased, the negative/positive association with reversion to NFG/progression to diabetes progressively strengthened (*P*-trend < 0.05).

**Table 3:** Multivariate Cox regression analysis of the role of AIP in assessing changes in glycemic status in patients with prediabetes

	Per 1000 person-years	HR (95%CI)			
		Non-adjusted Model	Model I	Model II	Model III
**Prediabetes to NFG**					
AIP	142	0.65 (0.59, 0.70)	0.75 (0.69, 0.82)	0.82 (0.75, 0.89)	0.89 (0.81, 0.98)
AIP (quartile)					
Q1	172	Ref	Ref	Ref	Ref
Q2	150	0.88 (0.82, 0.94)	0.94 (0.88, 1.01)	0.98 (0.91, 1.04)	1.01 (0.95, 1.08)
Q3	124	0.75 (0.70, 0.80)	0.83 (0.77, 0.89)	0.87 (0.81, 0.94)	0.92 (0.85, 0.99)
Q4	121	0.71 (0.67, 0.76)	0.80 (0.74, 0.86)	0.85 (0.79, 0.96)	0.91 (0.84, 0.98)
P-trend		< 0.0001	< 0.0001	< 0.0001	0.0034
**Prediabetes to Diabetes**					
AIP	53	2.07 (1.83, 2.35)	1.88 (1.65, 2.14)	1.67 (1.46, 1.91)	1.42 (1.24, 1.64)
AIP (quartile)					
Q1	35	Ref	Ref	Ref	Ref
Q2	49	1.42 (1.25, 1.61)	1.32 (1.16, 1.51)	1.28 (1.12, 1.46)	1.19 (1.04, 1.36)
Q3	60	1.80 (1.59, 2.04)	1.64 (1.45, 1.86)	1.55 (1.36, 1.76)	1.40 (1.23, 1.60)
Q4	68	1.96 (1.74, 2.21)	1.78 (1.58, 2.01)	1.63 (1.43, 1.85)	1.42 (1.24, 1.61)
P-trend		< 0.0001	< 0.0001	< 0.0001	< 0.0001

Abbreviations: HR: hazard ratios; CI: confidence interval; other abbreviations as in Table 1

Model I adjusted for age, sex

Model II adjusted for age, sex, SBP, LDL-C, ALT, BUN and Cr

Model III adjusted for age, sex, SBP, DBP, LDL-C, ALT, BUN, Cr, BMI, family history of diabetes, smoking status, drinking status

### Sensitivity analysis

To validate the stability of the association between AIP and either reversion or progression of prediabetes, we conducted association analyses under the WHO criteria, in the competing risk model, and among individuals without a family history of diabetes (Supplementary Table 2). The outcomes aligned with the primary analysis, indicating a negative association between AIP and reversion from prediabetes to NFG, and a positive association between AIP and progression from prediabetes to diabetes. In addition, based on the results from Model III, we computed the E-value to quantify the required magnitude of association between an unmeasured confounder and outcomes. The results showed that, in the negative correlation between AIP and the reversal of prediabetes, the point estimate of the E-value was 1.50. Comparing this with previously published results on factors reversing prediabetes [46–48], it seems unlikely that any unmeasured confounding factors would significantly affect the stability of our results. Moreover, in the positive correlation between AIP and the progression of prediabetes, the point estimate of the E-value was 2.19. This suggests that it is improbable for an unmeasured confounding factor to affect the result’s stability, given that 2.19 represents a relatively high degree of association.

### Visualization of the association between AIP and the reversion/progression of prediabetes

**Fig. 4:**
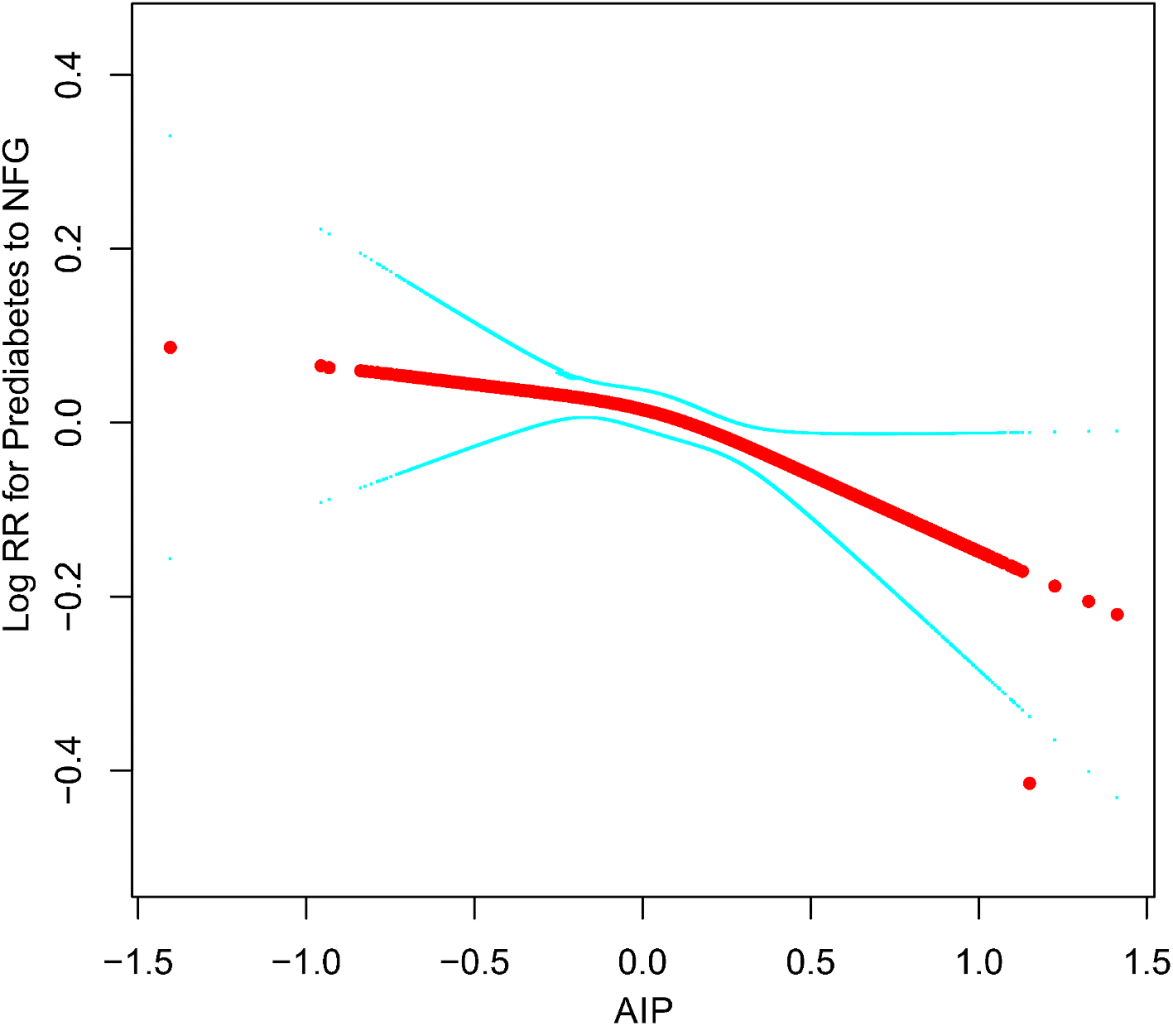
Apply the 4-knots RCS model to fit the dose-response curve of AIP and reversal of prediabetes to NFG. AIP: atherogenic index of plasma; NFG: normal fasting glucose; RCS: restricted cubic splines

**Fig. 5:**
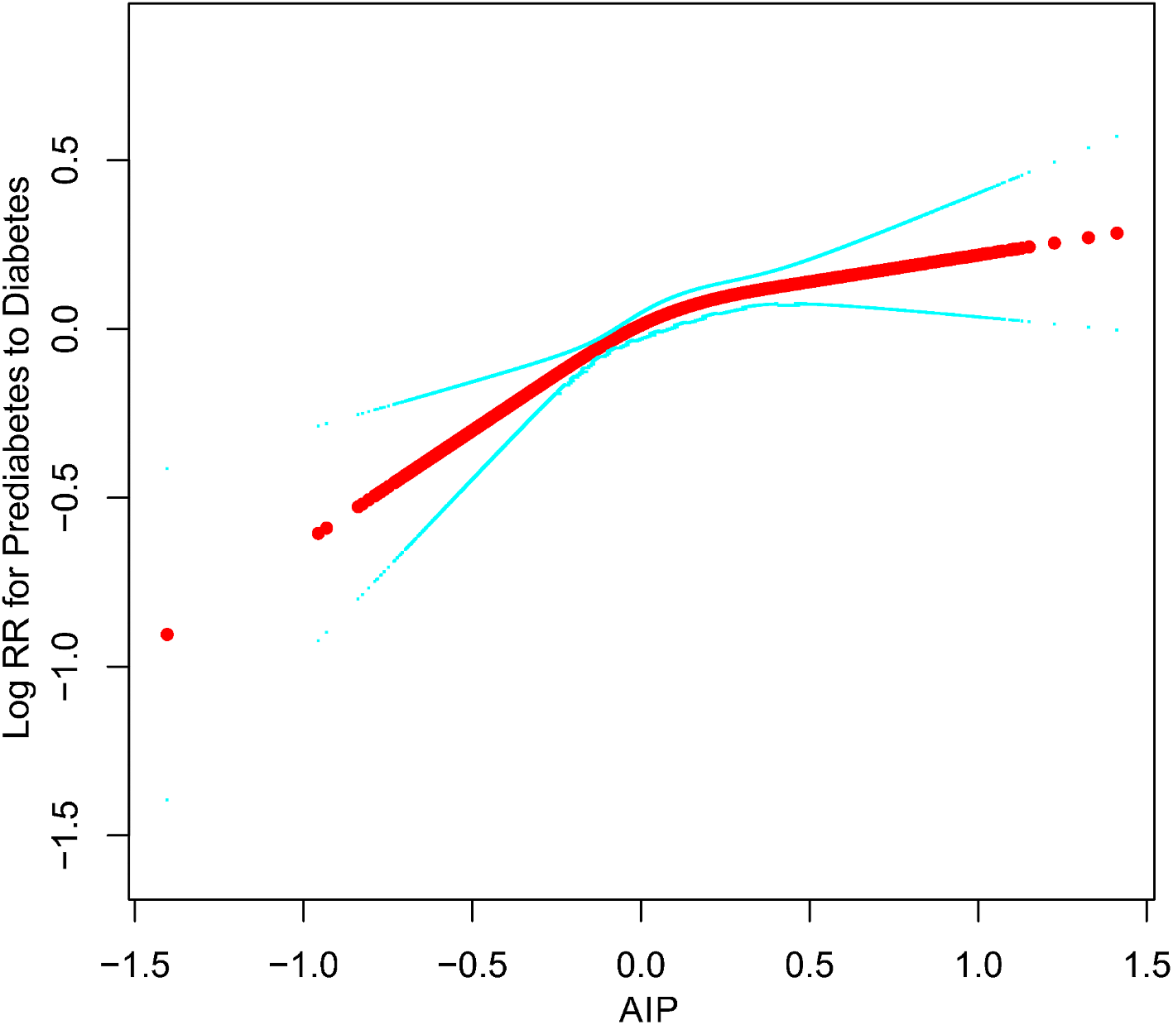
Apply the 4-knots RCS model to fit the dose-response relationship curve between AIP and progression from prediabetes to diabetes. AIP: atherogenic index of plasma; NFG: normal fasting glucose

Having established the association between AIP and the reversion/progression of prediabetes, we utilized RCS to further model and visualize the dose-response relationship between AIP and the aforementioned outcomes. After adjustments based on Model III, we observed that the relationships between AIP and either reversion or progression of prediabetes were not linear, with distinct inflection points evident (as depicted in Figs. 4 and 5). Using a recursive algorithm, we further identified an inflection point in the association of AIP with reversion to NFG at 0.04. Before this point, the curve indicating the association between AIP and reversion to NFG tended to be flat (HR 0.95, 95%CI:0.80–1.13); beyond this point, the negative association rapidly intensified (HR 0.84, 95%CI:0.72–0.99). Additionally, the inflection point for the association of AIP with progression from prediabetes to diabetes was calculated as 0.17. Prior to this point, the diabetes risk associated with AIP gradually escalated (HR 1.86, 95%CI:1.42–2.43); after which, the risk curve became relatively stable (HR 1.08, 95%CI:0.82–1.43) (as summarized in Table 4).

**Table 4:** The result of the two-piecewise Cox regression model

	HR (95%CI)	*P*-value
**Prediabetes to NFG**		
Fitting model by two-piecewise cox regression		
The inflection point of AIP	0.04	
<0.04	0.95 (0.80, 1.13)	0.5601
>0.04	0.84 (0.72, 0.99)	0.0327
**Prediabetes to Diabetes**		
Fitting model by two-piecewise cox regression		
The inflection point of AIP	0.17	
<0.17	1.86 (1.42, 2.43)	< 0.0001
>0.17	1.08 (0.82, 1.43)	0.5939

Abbreviations: HR: hazard ratios; CI: confidence

Adjusted for age, sex, SBP, LDL-C, ALT, BUN, Cr, BMI, family history of diabetes, smoking status, drinking status

### Subgroup analysis

In several of the most commonly observed phenotypic subgroups, we delved deeper into the association between AIP and the reversion/progression of prediabetes. As indicated in Table 5, within the sex, age, and BMI subgroups, we only observed an interaction between age and AIP in relation to the reversion/progression of prediabetes. Specifically, when compared to individuals aged ≥ 45 years, prediabetic patients aged < 45 exhibited a weaker negative association of AIP with reversion to NFG (HR: 0.94 vs. 0.82, *P*-interaction = 0.0010) and a stronger positive association with progression to diabetes (HR: 1.86 vs. 1.26, *P*-interaction = 0.0230).

**Table 5:** Exploratory subgroup analysis of the role and differences of AIP in assessing changes in glycemic status in prediabetes patients

	HR (95%CI)	
	Prediabetes to NFG	Prediabetes to Diabetes
**Sex**		
Male	0.88 (0.78, 0.98)	1.37 (1.16, 1.62)
Female	0.91 (0.79, 1.05)	1.56 (1.21, 2.00)
*P*-interaction	0.6844	0.4109
**Age, years**		
<45	0.94 (0.81, 1.08)	1.86 (1.38, 2.50)
≥ 45	0.82 (0.73, 0.93)	1.26 (1.07, 1.48)
*P*-interaction	0.0010	0.0230
**BMI, kg/m** ^**2**^		
<24	0.87 (0.76, 1.00)	1.95 (1.52, 2.52)
24-27.9	0.88 (0.76, 1.01)	1.28 (1.05, 1.57)
≥ 28	0.81 (0.63, 1.04)	1.34 (1.00, 1.80)
*P*-interaction	0.8505	0.0570

Models adjusted for the same covariates as in model III (Table 3), except for the stratification variable

Abbreviations: HR: hazard ratios; CI: confidence interval; other abbreviations as in Table 1

## Discussion

In this multi-center retrospective cohort study, we evaluated the role of AIP in determining the future glycemic outcomes of individuals with prediabetes. Our research indicated a positive correlation between AIP and the progression from prediabetes to diabetes, while a negative association existed between AIP and the reversion of prediabetes to NFG status. Furthermore, we observed a notable difference in the association between AIP and either reversion to NFG or progression to diabetes among different age subgroups. Specifically, the risk associated with AIP in relation to the progression from prediabetes to diabetes was relatively higher in the younger population; likewise, younger adults exhibited a more favorable association between AIP and the reversion of prediabetes to NFG.

Prediabetes is an intermediary stage between normoglycemia and diabetes [2]. Initially, the term “prediabetes” was primarily employed to identify individuals at high risk of developing diabetes in the future. However, as research has evolved, many studies have highlighted that this intermediate state doesn’t merely indicate the forthcoming risk of diabetes. It also inflicts significant detrimental effects on various organs/systems, amplifying the risk of microvascular diseases, macrovascular diseases, chronic kidney diseases, neuropathies, and even cancer [1–4]. It accelerates osteoporosis, brain aging, and even increases mortality risk [49, 50]. Fortunately, the hyperglycemic state of prediabetes is reversible. In the context of lifestyle intervention, evidence from the Malmö feasibility study shows that over 50% of prediabetic individuals reverted to normoglycemia within 6 years [19]; results from Saudi Arabia indicated a 52.1% reversion rate in prediabetic individuals within 18 months [16]; the Whitehall II cohort study revealed that 45% of individuals with impaired fasting glucose reverted to NFG within 5 years [11]; furthermore, the Daqing Diabetes Prevention study from China showed a 32.2% reversion rate within 6 years [8]. When pharmaceutical interventions were employed, evidence from the Canadian STOP-NIDDM cohort suggested that 35% of individuals with impaired glucose tolerance reversed normoglycemia within 1300 days post-acarbose treatment [15]; another study from the Indian Diabetes Prevention Program revealed a 40.9% reversion rate within 3 years after pioglitazone intervention [18]. While these pieces of evidence are scattered globally, they collectively echo the potent potential of prediabetes reverting to normoglycemia. In our current research with a median follow-up of 2.9 years, we observed that over 40% of prediabetic participants reversed their glucose levels to normal. Further, the cumulative incidence curve (Fig. 2) predicts that the reversion rate of prediabetes will continue to rise as time progresses.

Atherosclerotic lipid abnormalities are important modifiable risk factors for diabetes and cardiovascular events in diabetic patients. In recent years, intensified lipid management for these patients has gained increasing endorsement from endocrinologists and researchers alike [51, 52]. Notably, findings from the Steno-2 cohort study emphasized that the importance of lipid control in diabetic patients rivals that of glycemic control [53]. The AIP serves as a non-traditional lipid parameter for determining atherosclerosis risk. Its significant utility in the assessment and prediction of vascular-related diseases has been well-established [22, 23, 26, 27]. In our current study focusing on individuals in the prediabetic stage, we observed a significant positive correlation between AIP and diabetes onset. After thorough adjustment for confounders, our results indicated that for each unit increase in AIP, the risk of developing diabetes raised by 42% among prediabetic individuals. This discovery aligned with prior reports from studies centered on both Chinese populations and other ethnicities, suggesting that an elevated AIP augments the risk of diabetes onset [31–33, 54–58]. It’s imperative to highlight that the cohort in our study comprised of individuals in the prediabetic stage, setting it apart from earlier similar studies. Additionally, our investigation assessed the role of AIP in the regression of prediabetes to NFG and then found a negative correlation between AIP and such regression. Further analyses based on RCS revealed a non-linear relationship between AIP and both regression to NFG and progression to diabetes, with identified inflection points at 0.04 and 0.17, respectively. Interestingly, the AIP threshold for evaluating prediabetes regression was lower, suggesting stricter monitoring criteria might be needed for prediabetic individuals aiming to revert to NFG. From a therapeutic standpoint, we recommend that prediabetic patients maintain an AIP below 0.04, whereas for prevention, an AIP below 0.17 is advisable.

Subgroup analysis in our study yielded intriguing findings, revealing that age plays a pivotal role in the interaction with AIP concerning the regression or progression of prediabetes. More specifically, compared with individuals aged ≥ 45 years, those aged < 45 demonstrated a milder negative correlation between AIP and reversion to NFG, but a more pronounced positive correlation with the progression to diabetes. In layman terms, this suggests that AIP might be a more fitting metric for assessing the regression or progression of prediabetes in individuals below 45 years of age. A similar interaction between AIP and age was previously reported in another study evaluating the risk of prediabetes associated with AIP [31]. In a study conducted by Zheng et al., age was dichotomized at 60 years. They discerned that the prediabetic risk associated with AIP was markedly higher in individuals below 60 years compared to their counterparts above this age (HR: 1.56 vs. 1.11, *P*-interaction < 0.0001). Currently, a consensus regarding the superiority of AIP in assessing diabetes risk among younger demographics remains elusive. However, drawing on China’s societal context and policy nuances, we proffer some conjectures which may shed light on this phenomenon. It’s well-known that China implemented the One-Child Policy over several decades, leading to a drastic decline in the younger population, subsequently triggering a contraction in the nation’s labor force [59, 60]. However, despite this labor shrinkage, the relatively younger demographic remains integral to societal production. They are now grappling with augmented socio-psychological pressures compared to previous generations. This has significantly heightened their risk of lipid metabolic anomalies, including AIP, and glycemic disturbances, thereby amplifying their susceptibility to IR [61, 62].

For the relatively younger population, the advantage of using AIP to assess the reversion from prediabetes to NFG may be related to the following reasons: (1) As is widely known, the deficiency of functional β-cells is the primary cause of diabetes. For a long time, self-replication has been considered the main mechanism for β-cell maintenance and regeneration. However, the replication of β-cells declines rapidly with increasing age [63]. Therefore, being young implies a greater quantity and quality of β-cell regeneration [64]. The regeneration of β-cells will further improve lipid metabolism, including AIP, as well as glucose metabolism, leading to an advantage in reverting to NFG [65]. (2) Being young means having a higher β-cell recovery capacity [66, 67]. Therefore, subsequent improvements in AIP and reversion to NFG are more pronounced. (3) Lower body weight is noteworthy as it indicates a poorer reserve of pancreatic β-cells [68]. In the current study, compared to the population under 45 years of age, those aged ≥ 45 have a significantly lower body weight (70.34 kg vs. 68.71 kg). This lower body weight may be a significant factor in the age-related differences observed between AIP and the reversion to NFG.

### Strengths and limitations of the study

There are a few advantages worth mentioning: (1) The present study, for the first time, elucidates the association between AIP and the reversal from prediabetes to NFG. Additionally, it revalidates the critical role of AIP in risk assessment for the progression of diabetes within the prediabetic population. (2) After affirming the nexus between AIP and the prediabetes reversal, the study further identifies an inflection point associated with the reversal to NFG at 0.04, and another leading to the development of diabetes at 0.17. These threshold points play an essential role in both the therapeutic approach to prediabetes and the prevention of diabetes. (3) Compared to preceding studies on prediabetes reversal [22–28], the current research boasts an impressively extensive sample size. Furthermore, given its multi-centric nature, the evidentiary basis of this research can be considered relatively robust.

Limitations are similarly present in the current study, mainly as follows: (1) Given that the data originates from health examination institutions, it lacks oral glucose tolerance test information. Consequently, the outcomes of the current study are diagnosed based on FPG, which may underestimate the incidence rate of the outcomes. However, from another perspective, the diagnosis based on FPG alone in the current study may be more in line with the actual situation of group examinations for non-diabetic people in society, because non-diabetic people rarely receive oral glucose tolerance tests. (2) The relatively short follow-up duration might not comprehensively elucidate the relationship between AIP and the regression/progression of prediabetes. Further studies with extended follow-up periods are necessary for a more in-depth evaluation. (3) The evidence presented stems from medical examination data of a Chinese cohort; therefore, caution is advised when extrapolating these findings to other ethnicities. (4) To mitigate potential reverse causality, Li et al. initially excluded participants with a follow-up of less than two years. This resulted in a relative reduction of the overall sample size for the present study. (5) The current investigation solely assessed the association between baseline AIP and prediabetes regression/progression. Evaluating the longitudinal changes in AIP in relation to the regression or progression of prediabetes might further enhance the study’s value and significance, thereby warranting additional research. (6) The current study is based on a secondary analysis of a public data set, and the original data cannot be updated, thus inevitably leaving some unmeasured factors and potential residual confounding. As a workaround, we have calculated the E-value based on our final model to quantify the magnitude of association required by an unmeasured confounding factor. The results indicate that it is unlikely that there are unmeasured confounders that could affect the robustness of the results.

## Conclusion

This multi-center retrospective cohort study revealed a negative association between AIP and the regression of prediabetes to NFG, and underscored the pivotal role of AIP in assessing the risk of diabetes progression. According to threshold analysis, from a therapeutic perspective, maintaining AIP below 0.04 is crucial for prediabetic patients to revert to NFG. Preventatively, keeping AIP under 0.17 is significant for prediabetic individuals to mitigate the risk of diabetes onset.

### Electronic supplementary material

Below is the link to the electronic supplementary material.

**Supplementary Material 1:** Supplementary Figures 1–3

**Supplementary Material 2:** Supplementary Tables 1 and 2

## Data Availability

The data set supporting the results of this study has been uploaded to Dryad database.

## Abbreviations

AIP: atherogenic index of plasma
NFG: normal fasting glucose
SBP: systolic blood pressure
DBP: diastolic blood pressure
RCS: restricted cubic splines
BMI: body mass index
WHO: World Health Organization
ALT: alanine aminotransferase
AST: aspartate aminotransferase
HDL-C: high-density lipoprotein cholesterol
TC: total cholesterol
TG: triglyceride
LDL-C: low-density lipoprotein cholesterol
FPG: fasting plasma glucose
HR: hazard ratio
CI: confidence interval
IR: insulin resistance
Cr: creatinine
BUN: blood urea nitrogen
