# Supplementary material for: Evaluation of the role of atherogenic index of plasma in the reversion from Prediabetes to normoglycemia or progression to Diabetes: a multi-center retrospective cohort study
Source: Cardiovasc Diabetol. 2024 Jan 6;23:17. doi: 10.1186/s12933-023-02108-8 (PMC10771677; doi:10.1186/s12933-023-02108-8)
Supplement: Supplementary file 1 — Supplementary Material 1: Supplementary Figures 1–3 [file 12933_2023_2108_MOESM1_ESM.docx]

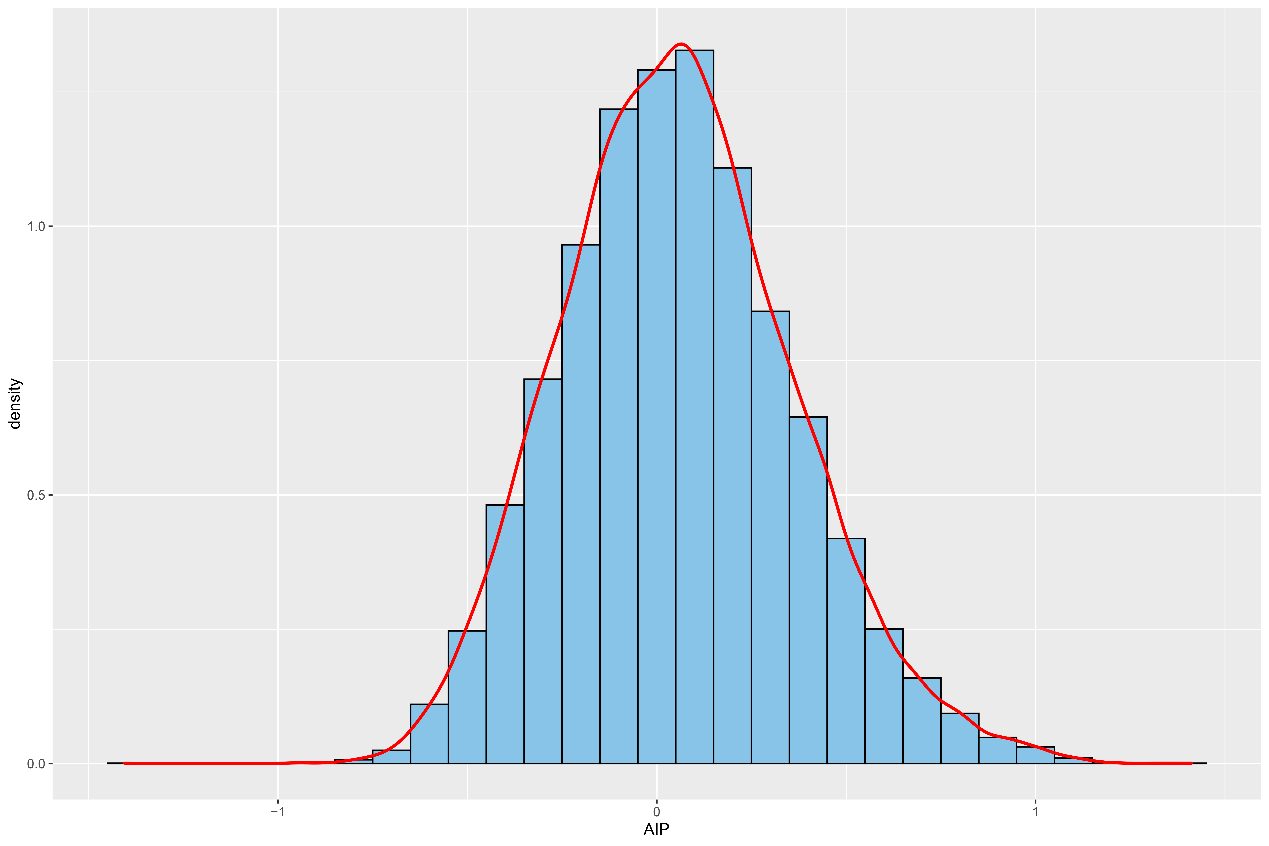


Supplementary Figure 1: Histograms show the population distribution of the AIP.


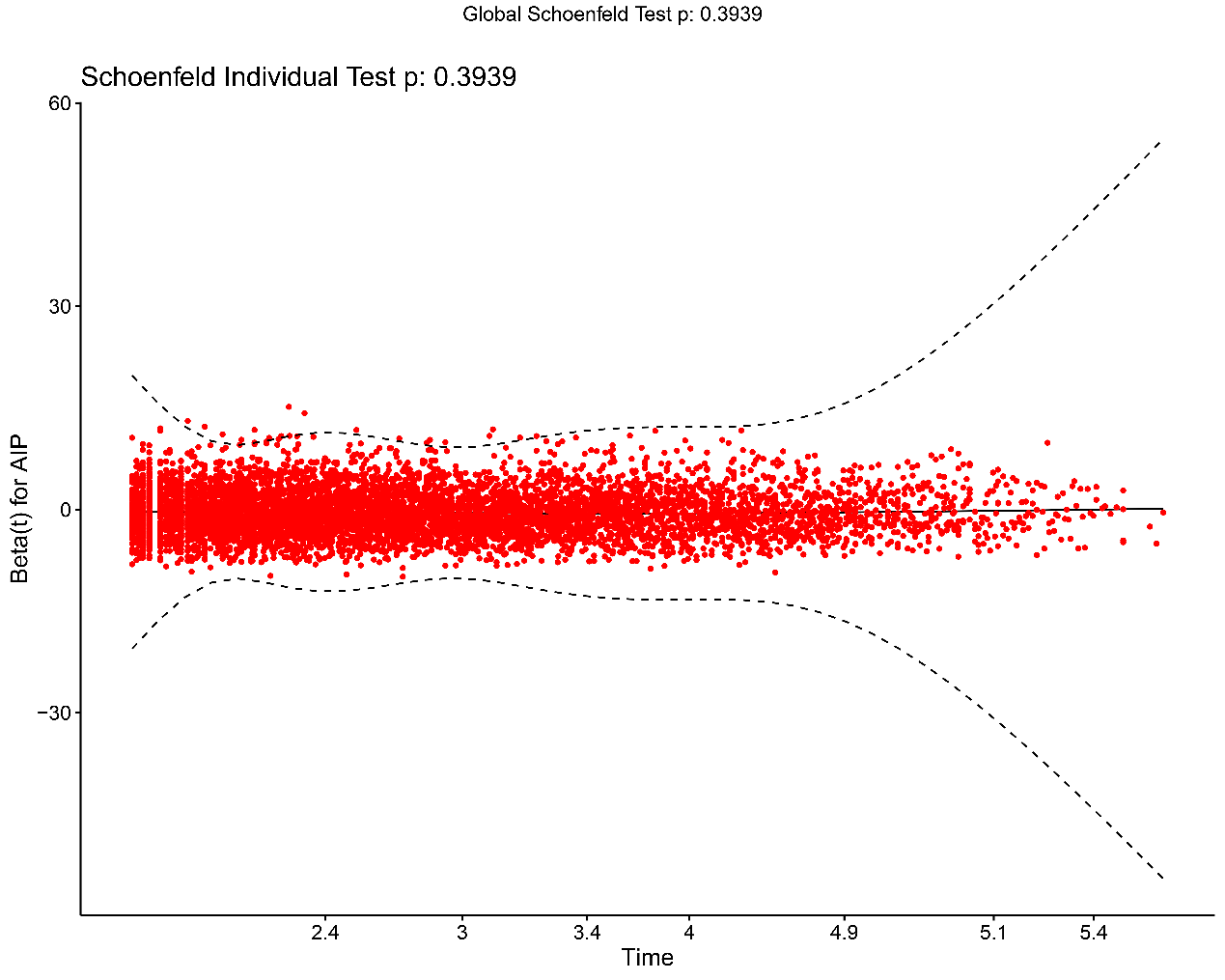


**Supplementary Figure 2**: Schoenfeld residual plot of AIP changes over time with reversion from prediabetes to NFG as the dependent variable. The p-value of Schoenfeld Residuals Test result is larger than 0.05 which indicated that AIP is not a time dependent variable and can be analyzed by Cox Proportional Hazards Model.


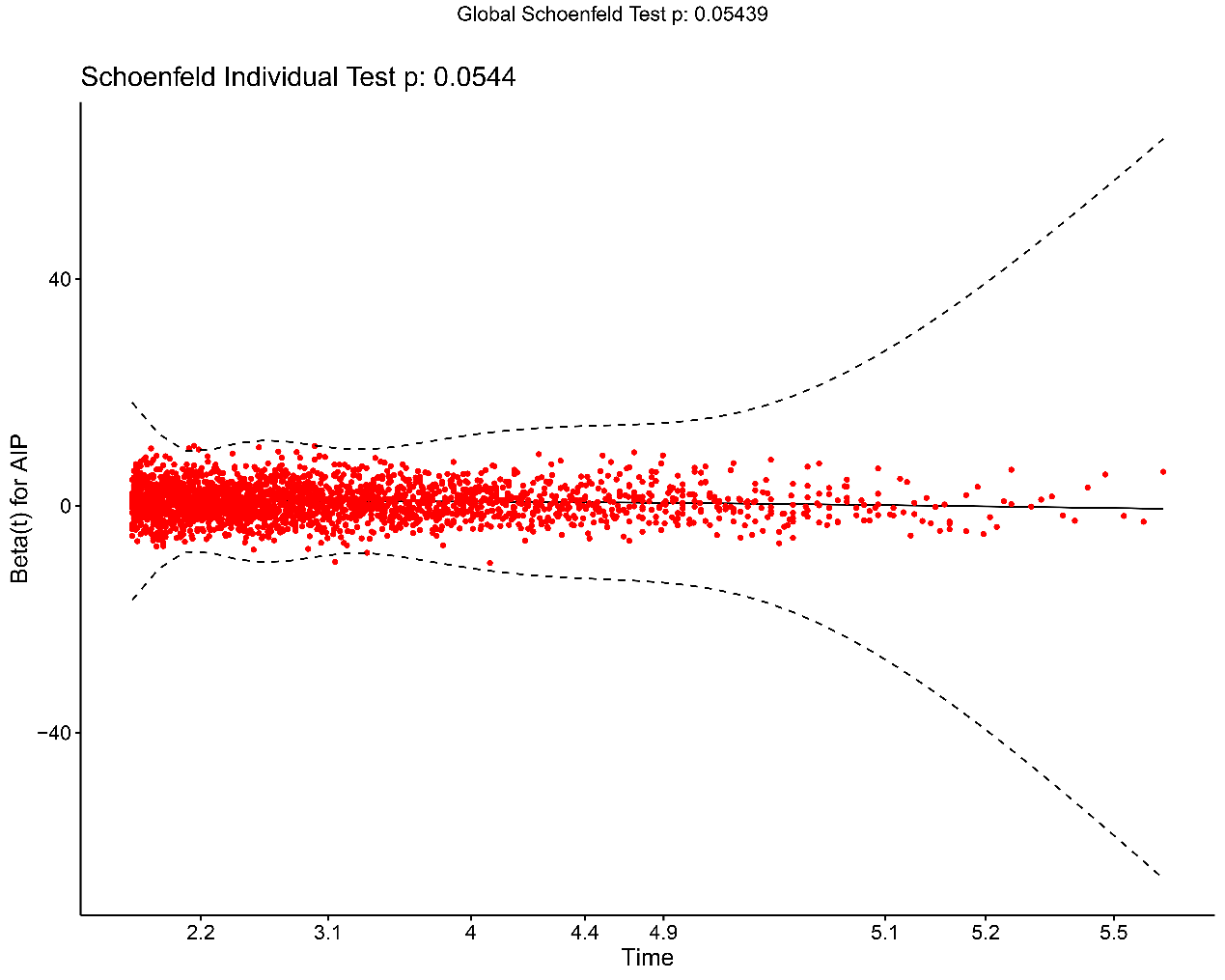


**Supplementary Figure 3:** Schoenfeld residual plot of AIP changes over time with progression from prediabetes to diabetes as the dependent variable. The p-value of Schoenfeld Residuals Test result is larger than 0.05 which indicated that AIP is not a time dependent variable and can be analyzed by Cox Proportional Hazards Model.
