# Supplementary material for: Evaluation of the role of atherogenic index of plasma in the reversion from Prediabetes to normoglycemia or progression to Diabetes: a multi-center retrospective cohort study
Source: Cardiovasc Diabetol. 2024 Jan 6;23:17. doi: 10.1186/s12933-023-02108-8 (PMC10771677; doi:10.1186/s12933-023-02108-8)
Supplement: Supplementary file 2 — Supplementary Material 2: Supplementary Tables 1 and 2 [file 12933_2023_2108_MOESM2_ESM.docx]

Supplementary Table 1: Diagnostic steps for collinearity between AIP and other covariates.

|  | VIF | | | |
| --- | --- | --- | --- | --- |
|  | Step 1 | Step 2 | Step 3 | Step 4 |
| AIP | 7.3 | 7.3 | 7.3 | 1.7 |
| Age | 1.4 | 1.4 | 1.4 | 1.4 |
| Sex | 2.6 | 2.6 | 2.6 | 2.6 |
| Height | 48.6 | 2.1 | 2.1 | 2.1 |
| Weight | 136 | NA | NA | NA |
| BMI | 79.9 | 1.3 | 1.3 | 1.3 |
| SBP | 1.9 | 1.9 | 1.9 | 1.9 |
| DBP | 1.7 | 1.7 | 1.7 | 1.7 |
| FPG | 1.1 | 1.1 | 1.1 | 1.1 |
| TC | 5.7 | 5.7 | NA | NA |
| TG | 5.6 | 5.6 | 5.1 | NA |
| HDL-C | 2.2 | 2.2 | 2.1 | 1.5 |
| LDL-C | 4.9 | 4.9 | 1.1 | 1.1 |
| ALT | 3.4 | 3.4 | 3.3 | 3.3 |
| AST | 3 | 3 | 3 | 3 |
| BUN | 1.1 | 1.1 | 1.1 | 1.1 |
| Cr | 1.7 | 1.7 | 1.7 | 1.7 |
| Family history of diabetes | 1 | 1 | 1 | 1 |
| Smoking status | 2.7 | 2.7 | 2.7 | 2.7 |
| Drinking status | 2.7 | 2.7 | 2.7 | 2.7 |

VIF: variance inflation factor; NFG: normal fasting glucose; VIF = 1/(1-R^2^). Abbreviations as in Table ​1.

Note: The variables with VIF>5 will be regarded as collinear variables and cannot be included in the multiple regression model.

Supplementary Table 2: Sensitivity analysis.

|  | No. of subjects |  | *P* value |
| --- | --- | --- | --- |
| Sensitivity-1 | 4,227 | HR (95%CI) |  |
| Prediabetes to NFG |  |  |  |
| AIP |  | 0.76 (0.60, 0.97) | 0.0252 |
| Prediabetes to Diabetes | |  |  |
| AIP |  | 1.27 (1.04, 1.54) | 0.0176 |
| Sensitivity-2 | 15,421 | SHR (95%CI) |  |
| Prediabetes to NFG |  |  |  |
| AIP |  | 0.49 (0.41, 0.59) | <0.0001 |
| Prediabetes to Diabetes | |  |  |
| AIP |  | 2.14 (1.73, 2.67) | <0.0001 |
| Sensitivity-3 | 14,611 | HR (95%CI) |  |
| Prediabetes to NFG |  |  |  |
| AIP |  | 0.89 (0.81, 0.98) | <0.0001 |
| Prediabetes to Diabetes | |  |  |
| AIP |  | 1.43 (1.23, 1.65) | <0.0001 |

SHR: subdistribution hazard ratios; CI: confidence interval; NFG: normal fasting glucose; other abbreviations as in Table 1.

Note 1: Models adjusted for the same covariates as in model III (Table 3).

Note 2: (1) Sensitivity-1: including 4,227 subjects according to WHO's diagnostic criteria for DM and IFG; (2) Results of Competing Risks Model Analysis (N=15,421); (3) sensitivity-3: excluding subjects with a family history of diabetes (N= 14,611)
